# Survey of malignant pleural mesothelioma treatment in Japan: Patterns of practice and clinical outcomes in tomotherapy facilities

**DOI:** 10.1093/jrr/rrab127

**Published:** 2022-02-09

**Authors:** Mikiko Nakanishi-Imai, Taro Murai, Masahiro Onishi, Atsuto Mouri, Takafumi Komiyama, Motoko Omura, Shigehiro Kudo, Akihiko Miyamoto, Masaru Hoshino, Shinichi Ogawa, Shizuko Ohashi, Masahiko Koizumi, Junichi Omagari, Hiroshi Mayahara, Katsuyuki Karasawa, Toshiyuki Okumura, Yuta Shibamoto

**Affiliations:** Department of Radiology, Nagoya City University Graduate School of Medical Sciences, Nagoya, 467-8601, Japan; Department of Radiology, Japanese Red Cross Aichi Medical Center, Nagoya Daini Hospital, Nagoya, 466-8650, Japan; Department of Radiology, Nagoya City University Graduate School of Medical Sciences, Nagoya, 467-8601, Japan; Oncology Center, Hidaka Hospital, Takasaki, 370-0001, Japan; Saitama Medical University International Medical Center Comprehensive Cancer Center, Department of Respiratory Medicine, Hidaka, 350-1298, Japan; Department of Radiology, Faculty of Medicine, University of Yamanashi, Chuo, 409-3898, Japan; Department of Radiation Oncology, Shonan Kamakura General Hospital, Kamakura, 247-8533, Japan; Department of Radiation Oncology, Saitama Cancer Center, Saitama, 362-0806, Japan; Hokuto Hospital Department of Radiation Therapy, Obihiro, 080-0833, Japan; Northern Fukushima Medical Center, Date, 960-0502, Japan; Department of Radiation Oncology Kizawa Memorial Hospital, Minokamo, 505-8503, Japan; Department of Radiology, Fukui-ken Saiseikai Hospital, Fukui, 918-8503, Japan; Department of Radiology, Nozaki Tokushukai Hospital, Daito, 574-0074, Japan; Department of Radiology, Koga Hospital 21, Fukuoka, 839-0801, Japan; Department of Radiation Oncology, Kobe Minimally-invasive Cancer Center, Kobe, 650-0046, Japan; Division of Radiation Oncology, Department of Radiology, Tokyo Metropolitan Cancer and Infectious Diseases Center Komagome Hospital, Tokyo, 113-8677, Japan; Department of Radiology, Mito Kyodo General Hospital, Mito, 310-0015, Japan; Department of Radiology, Nagoya City University Graduate School of Medical Sciences, Nagoya, 467-8601, Japan; Narita Memorial Proton Center, Toyohashi, 441-8021, Japan

**Keywords:** malignant pleural mesothelioma (MPM), tomotherapy, nationwide survey in Japan, whole hemithorax irradiation (WHI), intensity-modulated radiotherapy (IMRT), lung sparing pleural irradiation (LSPI), hyperthermia

## Abstract

We conducted a nationwide survey of tomotherapy for malignant pleural mesothelioma (MPM) in Japan. Fifty-six facilities were surveyed and data on 31 patients treated curatively between 2008 and 2017 were collected from 14 facilities. Twenty patients received hemithorax irradiation after extrapleural pneumonectomy (EPP) (first group). Five patients received irradiation without EPP (second group), while six received salvage radiotherapy for local recurrence (salvage group). Among the seven patients not undergoing EPP, five (four in the second group and one in the salvage group) were treated with lung sparing pleural irradiation (LSPI) and two with irradiation to visible tumors. Two-year overall survival (OS) rates in the first and second groups were 33% and 60%, respectively (median, 13 vs 30 months, *P* = 0.82). In the first and second groups, 2-year local control (LC) rates were 53 and 67%, respectively (*P* = 0.54) and 2-year progression-free survival (PFS) rates were 16% and 60%, respectively (*P* = 0.07). Distant metastases occurred in 15 patients in the first group and three in the second group. In the salvage group, the median OS was 18 months. Recurrence was observed in the irradiated volume in four patients. The contralateral lung dose was higher in LSPI than in hemithorax irradiation plans (mean, 11.0 ± 2.2 vs 6.1 ± 3.1 Gy, *P* = 0.002). Grade 3 or 5 lung toxicity was observed in two patients receiving EPP and hemithorax irradiation, but not in those undergoing LSPI. In conclusion, outcomes of EPP and hemithorax irradiation were not satisfactory, whereas LSPI appeared promising and encouraging.

## INTRODUCTION

Malignant pleural mesothelioma (MPM) is a rare neoplasm arising from the mesothelial surfaces of the pleural cavities and is caused by exposure to asbestos [1, 2]. The annual incidence of MPM worldwide is estimated to be approximately 28 000 cases per year [3], but is increasing, particularly in developing countries, due to the poor regulation of asbestos. Although the standard treatment for MPM remains controversial, surgical resection is recommended as first-line therapy [1, 4]. Surgical procedures for MPM are classified into three types: (i) partial pleurectomy (PP), (ii) pleurectomy/decortication (PD), and (iii) extrapleural pneumonectomy (EPP). PP is the partial removal of the parietal and visceral pleura, while PD is the complete removal of the gross tumor with parietal and visceral pleurectomy. EPP is the most invasive procedure, involving the *en bloc* resection of the pleura and adjacent normal tissues, such as the ipsilateral lung, pericardium and diaphragm [1, 2].

Radiotherapy effectively controls microscopic lesions. Therefore, the combination of EPP, chemotherapy and radiotherapy (the tri-modality strategy) is expected as radical treatment [1, 5]. Since MPM disseminates easily to the intrapleural cavity, whole hemithorax irradiation (WHI) after EPP generally covers from the lung apex to the diaphragm. The clinical target volume (CTV) has an irregular shape and is adjacent to radiosensitive organs. Intensity-modulated radiotherapy (IMRT) provides a conformal dose distribution to irregularly shaped targets, while minimizing the dose to radiosensitive organs. However, limited information is currently available on the clinical outcomes of IMRT for MPM.

The tomotherapy system (Accuray Inc., Sunnyvale, CA, USA) is a radiation delivery system that combines rotational IMRT and an imaging system [6]. The treatment field reaches 130 cm in the cranio-caudal direction. Due to these characteristics, tomotherapy is a suitable radiotherapy machine for MPM. However, these technical developments are sometimes a double-edged sword because the incidence of fatal pneumonitis may be higher with tomotherapy than with conventional radiotherapy [7]. Therefore, a nationwide survey and feedback on clinical outcomes were considered necessary. The present study was performed to evaluate the status quo in tomotherapy for MPM in Japan and provide insights that will improve outcomes.

## METHODS

### Data collection and patient selection

A nationwide survey of patients with MPM treated with tomotherapy between 2008 and 2017 was conducted. The first questionnaire was sent to 57 facilities in Japan, which had the tomotherapy system. A more detailed questionnaire was then sent to facilities with treatment experience of MPM. To analyze the outcomes of curative treatment, information on patients fulfilling the following criteria was collected from these facilities: (i) pathologically proven MPM, (ii) clinical stage III or lower MPM, (iii) WHO performance status ≤2, and (iv) prescribed radiation dose ≥40 Gy. Exclusion criteria were: (i) multiple malignant tumors, and (ii) previous irradiation to the chest.

The following data on patients were collected: (i) age, (ii) sex, (iii) clinical history, (iv) history of exposure to asbestos, (v) pathological subtypes, (iv) stage, (v) surgery date, procedure and the residual tumor status, (vi) chemotherapy and other treatment history, and (vii) radiotherapy details, schedules and planning data. Information on the targets and normal organs as well as the dose distribution was collected using the Digital Imaging and Communications in Medicine (DICOM)-RT format. These planning data were captured and analyzed in the radiotherapy treatment planning system, RayStation (v.10, RaySearch Laboratories, Sweden).

Clinical outcome data comprised: (i) the date of death, (ii) last follow-up date, (iii) date of recurrence in the irradiated area, (iv) date of recurrence in any lesion, and (v) early (≤ 90 days after irradiation) and late (> 90 days) toxicities. The locations of recurrence and metastases were requested. Toxicities were evaluated according to Common Terminology Criteria for Adverse Events version 4.0. Disease stages were classified according to the UICC TMN 8^th^ edition.

The primary endpoint was the 2-year overall survival (OS) rate, as assessed from the first day of the MPM treatment. The secondary endpoints were the 2-year local control (LC) and 2-year progression-free survival (PFS) rates assessed from the first day of radiation to the date of local recurrence or distant metastasis. LC failure was defined as recurrence in the irradiated field.

All patients’ data was anonymized. The present study was approved by the Institutional Review Board at each facility and registered in the UMIN clinical trial database (No, UMIN000042430).

### Planning evaluation

CTV was categorized to: (i) the volume covered by WHI, (ii) the volume covered by lung sparing pleural irradiation (LSPI), and (iii) visible tumors only. WHI covered the whole hemithorax from the thoracic inlet to the insertion of the diaphragm, along the ribs laterally and along the mediastinal pleura, pericardium and hilum medially. CTV in LSPI was defined as gross visible tumors and the parietal and visceral pleura.

To evaluate the plan quality of WHI and LSPI, the minimum dose of 95% of the planning target volume (PTV) (D95%) and the dose distribution to organs at risk were evaluated using RayStation. The conformity index (CI) and uniformity index (UI) were calculated according to the following formulae [8].

(1) \begin{equation*} \mathrm{UI}=\mathrm{D}5\%/\mathrm{D}95\% \end{equation*}

(2) \begin{equation*} \mathrm{CI}=\left({\mathrm{V}}_{\mathrm{PTV}}\ast{\mathrm{V}}_{\mathrm{TV}}\right)/{\left({\mathrm{TV}}_{\mathrm{PV}}\right)}^2 \end{equation*}

where V_PTV_ = PTV (cc), TV_PV_ = lesion volume (cc) covered by the prescribed isodose, V_TV_ = prescribed isodose volume (cc) and D5% = minimum dose delivered to 5% of the PTV. Lower CI indicates higher conformity, and lower UI indicates better homogeneity. Ideal CI and UI are both 1.

To standardize normal organ doses, the lung, heart, liver, kidneys, esophagus and spinal cord were re-contoured by 1 planner (M.NI). The mean lung dose was defined as the cumulative dose per the remaining total lung volume. VXGy was the volume percentage of the organ covered with X Gy.

### Statistical analysis

OS, LC and PFS rates were calculated using the Kaplan–Meier method; LC and PFS were assessed by accounting for death as a competing risk. The survival of each subgroup patient was tested with the Log-rank test. The Gray test was applied to evaluate the LC and PFS outcomes of each subgroup. Differences in patient and treatment characteristics and toxicities were examined by Fisher’s exact test or student’s t-test (for continuous variables). All statistical analyses were performed with Easy R (EZR) (Saitama Medical Center, Jichi Medical University, Saitama, Japan), which is a graphical user interface for R (The R Foundation for Statistical Computing, Vienna, Austria).

## RESULTS

### Patient selection and data cleaning

**Fig. 1.**
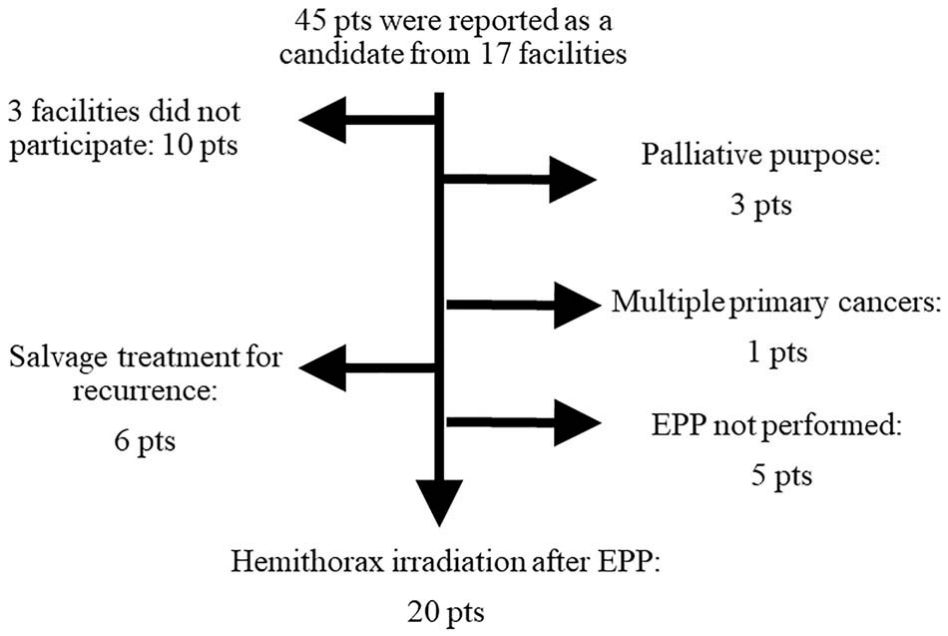
Patient selection flow chart. EPP, extrapleural pneumonectomy; pt, patient.

In the nationwide survey of MPM patients, 56 out of 57 facilities (98%) answered the first questionnaire. Twenty out of the 56 facilities had treated 63 MPM patients in the time period indicated. Of these patients, 45 were prescribed to receive ≥40 Gy with curative intent. At this stage, four facilities decided not to participate in this survey because of in-house regulations or the loss of planning data. Therefore, the clinical data of 31 patients from 14 facilities (two academic and 12 community hospitals) were ultimately analyzed. Twenty patients underwent EPP and received hemithorax radiotherapy (first group), five received radiotherapy without surgery (second group) and six were irradiated as salvage treatment for local recurrence (salvage group). Patient selection and characteristics are shown in Fig. 1 and Table 1, respectively. Two academic and two community hospitals had treatment experience of three or more patients. One community hospital treated six patients after EPP as first-line treatment and two as salvage treatment. Two academic hospitals treated four patients each. In another community hospital, three underwent tomotherapy. All second group patients and four of the five LSPI patients underwent radiotherapy in these experienced facilities. In the second group patients, reasons for not undergoing EPP were difficulty of peeling and *en bloc* resection in three, uncontrollable diabetes mellitus in one, and refusal of EPP in one.

**Table 1:** Patient and treatment characteristics

		First group	Second group	Salvage	*P*-value
Patient Number		20	5	6	
Age (mean ± SD) (years)		62 ± 7.7	66 ± 11	62 ± 9.7	0.68
Age (years)	(>65: ≤65)	10: 10	4: 1	3: 3	0.51
Male: Female		19: 1	3: 2	5: 1	0.07
WHO PS	(0: 1: 2)	9: 9: 2	3: 1: 1	1: 4: 1	0.47
Tumor site	(Right: Left)	7: 13	5: 0	2: 4	0.06
Gross residual tumors (+: -)		3: 17	5: 0	4: 2	< 0.001
Pathology					
(Epi: Bip: Sar: unknown)		13: 7: 0: 0	2: 2: 1: 0	3: 0: 2: 1	0.03
Asbestos exposure					
(+: -: unknown)		11: 5: 4	1: 3: 1	1: 3: 2	0.97
Stage (1a: 1b: 2: 3a: 3b)		0: 12: 1: 5: 2	1: 3: 0: 0: 1	1: 1: 2: 1: 1	0.16
T stage	(1: 2: 3: 4)	0: 9: 9: 2	1: 1: 2: 1	1: 0: 4: 1	0.37
N stage	(0: 1)	13: 7	5: 0	3: 3	0.56
Surgery type	(EPP: PD: None)	20: 0: 0	0: 0: 5	4: 1: 1	< 0.001
Chemotherapy					
(None: Con: Adj: NAC)		5: 0: 4: 11	2: 1: 1: 1	0: 0: 3: 3	0.17
Hyperthermia (+: -)		5: 15	1: 4	2: 4	1
Irradiated volume					
(WHI: LSPI: visible tumor only)		20: 0: 0	0: 4: 1	4: 1: 1	< 0.001

Adj: adjuvant, Bip: biphasic, Con: concurrent chemotherapy, Epi: epithelioid, NAC: neo-adjuvant chemotherapy, LSPI: lung sparing pleural irradiation, PS: performance status, PD: pleurectomy/decortication, Sar: sarcomatoid, SD: standard deviation, WHI: whole hemithorax irradiation.

**Table 2:** Radiotherapy details

		*WHI*	*LSPI*	*P-value*
Patient Number (Mean ± SD)		24	5	
PTV(cc)		3583 ± 1141	2640 ± 1037	0.1
Prescription dose (Gy) (median, range)		50.4 (45–60)	45 (45–60)	0.71
Fraction number (median, range)		28 (25–33)	15 (15–30)	0.1
Conformity Index		1.96 ± 0.95	2.62 ± 1.09	0.18
Uniformity index		1.08 ± 0.05	1.37 ± 0.18	< 0.001
CTV covered				
	Surgical tract (+: -)	7: 17	2: 3	0.46
	Mediastinal region (+: -)	11: 13	3: 2	0.32
Tumor side (right: left)		7: 17	5: 0	0.001
Virtual block (+: -)		4: 20	1: 4	1
Mean lung dose (Gy)		6.1 ± 3.1	18.4 ± 2.8	< 0.001
Mean heart dose (Gy)		29.5 ± 8.5	25.4 ± 4.4	0.64
Spinal cord maximum dose (Gy)		29.5 ± 2.4	39.3 ± 4.5	0.15
Esophagus maximum dose (Gy)		53.4 ± 1.5	45.6 ± 3.7	0.27
Stomach maximum dose (Gy)		50.1 ± 3.0	30.2 ± 2.1	0.02

LSPI: lung sparing pleural irradiation, PTV: planning target volume, SD: standard deviation.

WHI: whole hemithorax irradiation.

The UI and mean lung dose were significantly higher in LSPI plans (*P* < 0.001).

The median age of patients was 65 years (range: 44–76 years) in the first group, 66 years (49–76 years) in the second group and 66 years (43–68 years) in the salvage group. Twenty-seven patients (87%) were male. The median interval between surgery and radiotherapy was 5 months (range: 0–7 months) in the first group. Twenty-four patients underwent EPP. No patient underwent PP. Five patients in the first group and one in the second group received hyperthermia using a previously reported technique [9, 10]. In the salvage group, two received hyperthermia as the first-line treatment combined with EPP. Twenty-four patients received chemotherapy (pemetrexed plus cisplatin in 20 and pemetrexed plus carboplatin in three) [11]. Chemotherapy agents were unclear in one patient.>

### OS, PFS and LC rates

**Fig. 2.**
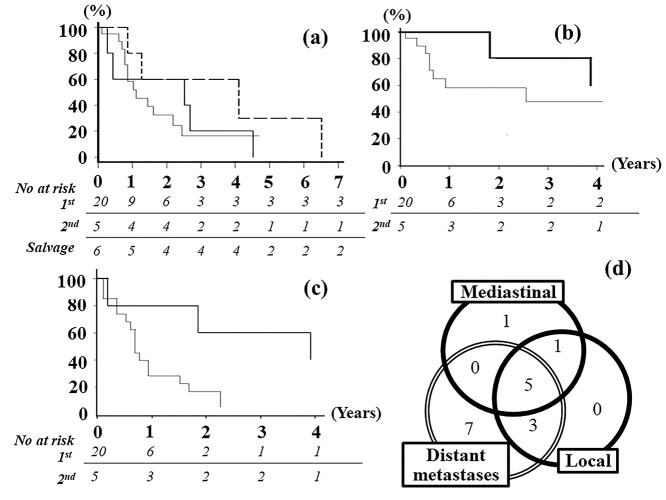
(**a**) OS from the first treatment. (**b**) LC from radiotherapy. (**c**) PFS from radiotherapy. (**d**) Patterns of recurrence in the first group. Thin line, first group; bold line, second group; dotted line, salvage group.

Two-year OS rates after the first treatment in the first and second groups were 33% and 60%, respectively (Fig. 2a, median OS period, 13 vs 30 months, *P* = 0.82). In these two groups, survival was slightly longer in female patients (54 vs 16 months, *P* = 0.06). OS rates did not significantly differ with the use of hyperthermia (+ vs -, 24 vs 16 months, *P* = 0.76) or chemotherapy (+ vs -, 16 vs 30 months, *P* = 0.82). Furthermore, no significant differences were observed according to stage (stage I or II vs III), T-stage (T1–2 vs T3 vs T4), lymph node metastasis (+ vs -), pathological findings (epithelioid vs others), facility treatment experience (≥ 3 vs < 3 cases), performance status (0 vs 1 vs 2), exposure to asbestos (+ vs – vs unknown), or age (> 65 vs ≤ 65) (*P* ≥ 0.26) (Supplemental materials, Table S1). Two-year LC rates in the first and second groups were 53% and 67%, respectively (Fig. 2b, *P* = 0.54). Two-year PFS rates in the first and second groups were 16% and 60%, respectively (Fig. 2c, *P* = 0.07). LC and PFS rates were significantly better in patients with a lower T or TMN stage or without lymph node metastasis (*P* < 0.05) (Supplemental materials, Figs S1 and S2). Regarding the gross residual tumor status or CTV coverage of the surgical tract or mediastinal region, no significant differences were noted in the OS, LC, or PFS (*P* ≥ 0.25).

Recurrence was observed in 17 patients in the first group and three in the second group and occurred early after irradiation (median, 8 months). The patterns of recurrence in the first group are shown in a Venn diagram (Fig. 2d). The most frequently observed recurrence pattern was distant metastases (15 cases) followed by regional lymph nodes (seven cases) in this group. Distant metastases were detected in the contralateral lung in nine patients and the peritoneum in four. Metastases to the 8^th^ contralateral costal bone and para-aortic lymph nodes were noted in one case each. Peritoneal and lung metastases simultaneously occurred in one patient. In the second group, distant metastases occurred in three cases; the sites of recurrence were the lungs, peritoneum and brain, respectively. Mediastinal recurrence simultaneously occurred in two cases in this group. Thirteen out of 18 cases in the first and second groups died within 12 months (median, 5.5 months) after the development of distant metastases.

In the salvage group, the interval from the first treatment to irradiation was 23 months (range, 16–149 months). Median OS after the first treatment was 67.5 months (27–170 months, Fig. 2a). On the other hand, median OS after irradiation was 18 months (range, 9–78 months). In this group, 2-year LC and PFS rates were 44% and 33%, respectively. In contrast to the first and second groups, local recurrence was observed in four cases.

### Radiotherapy details

**Fig. 3.**
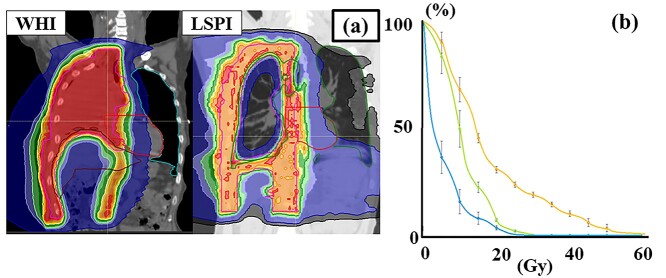
Typical dose distributions and dose volume histogram of WHI and LSPI. (**a**) Dose distributions of WHI and LSPI plans. (**b**) The yellow line is a dose volume histogram of the lung in LSPI plans, green is that of the contralateral lung in LSPI and light blue is that of the contralateral lung with WHI. Each line shows the average dose of each plan. Bars show standard errors at the dose point.

**Fig. 4.**
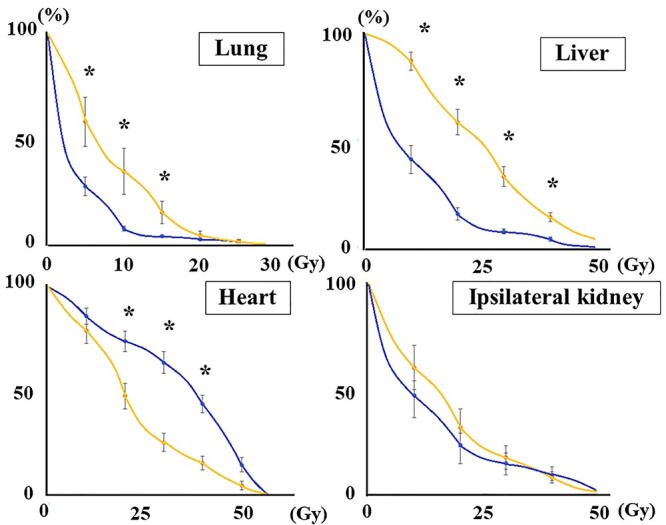
Dose volume histogram of WHI. Blue lines represent the average dose volume histogram in patients with left-side tumors, and yellow lines for those with right-side tumors. Bars show standard errors at the dose point. * indicates a significant difference between the right and left sides.

Twenty-four patients were treated with WHI and five with LSPI (Table 2); typical dose distributions are shown in Fig. 3. In the WHI plan, the median prescribed dose was 50.4 Gy/28 fr. Regarding gross residual tumors, 6 Gy–10 Gy was added using boost irradiation or a simultaneous integrated boost. Two patients did not complete radiotherapy at 9 and 19.8 Gy, respectively. The dose-volume parameters of normal tissues in the WHI plan are summarized in Fig. 4. In patients with left-side tumors, the heart received higher doses (Fig. 4, V20-40Gy, *P* < 0.005). Themean heart dose was 20.8 ± 6.9 Gy in patients with right-side tumors and 33 ± 6.4 Gy in those with left-side tumors (*P* < 0.001). Higher doses were delivered to the lung (V5-15Gy, *P* < 0.05) and the liver (V10-40Gy, *P* < 0.001) in patients with right-side tumors (Fig. 4). The V5Gy of the contralateral lung was higher than 50% in six patients and 60% in three. CI and UI did not significantly differ between experienced facilities with ≥3 cases and others (*P* > 0.51).

**Table 3:** Toxicity and patient number

Early toxicities (grade)		1	2	3	4	5	≥ 2	*P*-value
WBC decrease	WHI	2	4	1			5	0.24
	LSPI	1		1			1	
Anemia	WHI	11	2				2	0.13
	LSPI	1	1	1			2	
PC decrease	WHI	5	1	1			2	1
	LSPI	1						
Pneumonitis	WHI	1		1		1	2	1
	LSPI	1	1				1	
Dyspnea	WHI	7	1	1			2	1
	LSPI	2						
Hypoxia	WHI	3		1			1	1
	LSPI							
Esophagitis	WHI	5	4				4	1
	LSPI	2	1				1	
Nausea	WHI	7	4				4	0.55
	LSPI	2	1				1	
Fatigue	WHI	7	4	1			5	1
	LSPI	2						
Dermatitis radiation	WHI	10	1				1	0.32
	LSPI		1				1	
Anorexia	WHI	3						1
	LSPI							
Chest wall pain	WHI	2	1				1	1
	LSPI							
Cough	WHI	1						1
	LSPI							
Gastritis	WHI	1						1
	LSPI							
***Late toxicities (grade)***		***1***	***2***	***3***	***4***	***5***	≥ 2	***P*-value**
Pneumonitis	WHI	1						1
	LSPI		1				1	
Dyspnea	WHI	9						1
	LSPI		1				1	
Hypoxia	WHI	2						1
	LSPI							
Pericardial effusion	WHI	1						1
	LSPI							

LSPI: lung sparing pleural irradiation, PC: platelet count, WBC: white blood cell count, WHI: whole hemithorax irradiation.

Although CI did not significantly differ between WHI and LSPI (1.96 ± 0.94 vs 2.62 ± 1.09, *P* = 0.18), WHI target doses were more homogeneous than those of LSPI (UI, 1.08 ± 0.05 vs 1.37 ± 0.18, *P* < 0.001). Fig. 3b shows the lung doses of the LSPI and WHI plans. The mean lung dose was higher in LSPI than in WHI (18.3 ± 2.8 Gy vs 6.1 ± 3.1 Gy, *P* < 0.001). The contralateral lung dose in LSPI was higher than that in hemithorax irradiation plans (mean, 11.0 ± 2.2 Gy vs 6.1 ± 3.1 Gy, *P* = 0.02). The V5Gy of the contralateral lung was higher than 50% in all LSPI patients (Fig. 3b, green line).

## TOXICITIES

Toxicities after radiotherapy are summarized in Table 3. Grade 3 and grade 5 pulmonary toxicities were observed in one patient each. These patients were treated with EPP and WHI, respectively. Their V5Gy of the contralateral lung were 100% and 37%, respectively. The V20Gy were 17.1% and 5.5%, respectively. Although the lung dose was higher in LSPI than in WHI, grade 3 pneumonitis was not observed in these LSPI patients. In total, grade ≥ 2 pulmonary toxicities occurred in five. Although dose blocking structures (virtual block) were contoured in five of these LSPI or WHI patients, the contralateral lung dose did not differ significantly (*P* > 0.46). However, patients planned with the virtual block did not suffer from grade ≥ 2 toxicity. Grade 2 or higher cardiac toxicity was not detected in 31 patients.

## DISCUSSION

The treatment of MPM remains challenging due to its rarity and highly malignant potential. Based on randomized trials, systemic therapy is the only proven intervention that improves survival in MPM [11–13]. The most divisive topic is the role of local treatment. A randomized trial (MesoVATS) did not show any benefit of PP for OS (1-year OS, 52% in the PP group vs 57% in the control group) [14]. In our nationwide survey, no patient underwent PP, while EPP was widely adopted. Although LC appeared to be achievable in patients undergoing WHI and EPP, PFS and OS rates were not satisfactory. Distant metastases frequently occurred after irradiation. Most patients died within 12 months of the development of distant metastasis. These unfavorable outcomes may be caused by the high incidence of distant metastasis and its lethality. This survey suggested that various combinations of surgical resection, radiotherapy and hyperthermia were performed for MPM at facilities with experience of two MPM cases or less. Therefore, a nationwide survey to study MPM is needed to obtain a higher level of evidence. In addition, this survey will provide insights into the treatment of MPM.

EPP and WHI have been expected to improve OS for a decade [1, 5]. However, the findings of recent randomized trials in Europe contradict this expectation. In the MARS trial, 50 patients were randomized to compare the tri-modality strategy with chemotherapy alone [15]; 1-year OS rates were worse in the tri-modality group than in the chemotherapy group (52% vs 73%). SAKK 17/04 is a randomized study that evaluated the efficacy and safety of the tri-modality strategy [16, 17], and showed that LC did not significantly differ between the no radiotherapy and radiotherapy groups (median, 7.6 and 9.4 months, respectively). The findings of these trials remain controversial due to the insufficient quality control of the intervention and the small number of participants [18, 19]. In our survey, the salvage group survived for 9–78 months after irradiation. This result was similar to that for the other 2 groups. Therefore, the grade of malignancy in this disease widely varies and more than 100 patients are required for sufficient randomization. As such, any conclusion based on a small number of participants may be invalid, as stated by some surgeons [18, 19]. However, we consider the tri-modality strategy to still be unacceptable. The perioperative mortality of EPP is 5–10%, even by experienced surgeons in high volume centers [20]. In addition, few patients complete these three interventions. In the SAKK 17/04 study, one third of 151 patients was not eligible for radiotherapy, and an additional one third was excluded before randomization [16, 17]. Therefore, only 54 patients were randomly assigned to WHI or none. In the Japan Mesothelioma Interest Group 0601 trial, the tri-modality protocol was only completed by 41% patients because of treatment-related complications despite low perioperative mortality [5]. In our survey, 10% of patients who tolerated EPP were unable to complete WHI.

Grade 4 and 5 radiation pneumonitis occurred in two out of 27 patients (7%) in the SAKK 17/04 study, which used conventional radiotherapy. Early series of WHI for MPM using IMRT resulted in a high incidence of fatal pneumonitis. Allen *et al.* [7] reported six cases of fatal pneumonitis in 13 patients treated with IMRT after EPP. The V5Gy of the contralateral lung and mean lung dose were higher in patients who developed pneumonitis than in those who did not. Kristensen *et al.* [21] analyzed the dose-volume metrics of 26 patients, including four with grade 5 lung toxicity. In our survey, grade 3 and 5 lung toxicities were observed in one patient each undergoing EPP. These results indicate that the contralateral lung after EPP is more sensitive to low dose irradiation than the normal lung receiving usual thoracic radiotherapy. The treatment burden of EPP was not negligible, and we do not recommend the routine delivery of the combination of EPP and WHI.

Based on these findings, PP or EPP for MPM patients is not recommended. PD was previously considered to be an option for surgical cytoreduction. A randomized trial in the UK, the MARS-2 trial (NCT02040272) is ongoing to compare PD with no surgery in MPM patients. Furthermore, LSPI is being developed as a new cytoreduction measure in radiation oncology. Many facilities have so far been reluctant to use LSPI. In our nationwide survey, five patients received LSPI. Although four of the five patients in the second group were unfit to EPP, LC, PFS and OS were comparable to those of patients with EPP. The outcome may be encouraging. In a phase II study conducted by Rimner *et al.* [22–25], 27 patients were treated with PD and LSPI; their median PFS and OS were 12.4 and 23.7 months, respectively, with grade 3 pneumonitis developing in two. Patel *et al.* [26] summarized seven clinical trials. The incidence of grade 3 pneumonitis ranged between 0 and 16%. Grade 4 or 5 pneumonitis was observed in less than 1.5% of patients. Based on these results, a randomized trial (NRG LU-006) comparing PD alone and PD plus LSPI has been initiated. In our survey, grade 3 or higher pulmonary toxicity was not observed in five patients receiving LSPI, despite that the lung dose was higher in LSPI than in WHI. This may be because extensive surgery reduces tolerance to radiation. In our survey, patients with a virtual block did not suffer from grade ≥ 2 pulmonary toxicity. Generally, using a virtual block can reduce the contralateral lung dose [6, 8]. The difference in the contralateral lung dose was not significant in this survey probably due to the small sample size. At least, the planners who used the virtual block paid more attention to the contralateral lung dose than those who did not. Considering these, even in LSPI planning, contralateral lung dose reduction and usage of a virtual block may be recommended. Regardless of the findings of MARS-2 and NRG LU-006, this approach may be an option for MPM patients unable to tolerate surgery and chemotherapy.

In conclusion, EPP, hemithorax irradiation and other various interventions were adopted for MPM in tomotherapy facilities. Although tri-modality outcomes are not satisfactory, LSPI outcomes appeared promising and encouraging. Further nationwide surveys are warranted to obtain a higher level of evidence.

## Supplementary Material

Suppl_legend1101_rrab127

TableS1_rrab127

## CLINICAL TRIAL REGISTRATION NUMBER

UMIN clinical trial database (No, UMIN000042430).
