# Supplementary material for: Survey of malignant pleural mesothelioma treatment in Japan: Patterns of practice and clinical outcomes in tomotherapy facilities
Source: J Radiat Res. 2022 Feb 9;63(2):281–9. doi: 10.1093/jrr/rrab127 (PMC8944311; doi:10.1093/jrr/rrab127)
Supplement: Suppl_legend1101_rrab127 [file suppl_legend1101_rrab127.docx]

**Supplemental materials legends**

**Figure S1. Local control curves from radiotherapy**

Epi: epithelioid

**Figure S2. Progression-free survival curves from radiotherapy**
