# Supplementary material for: Survey of malignant pleural mesothelioma treatment in Japan: Patterns of practice and clinical outcomes in tomotherapy facilities
Source: J Radiat Res. 2022 Feb 9;63(2):281–9. doi: 10.1093/jrr/rrab127 (PMC8944311; doi:10.1093/jrr/rrab127)
Supplement: TableS1_rrab127 [file tables1_rrab127.docx]

**Table S1. Univariate analyses of OS, LC and PFS**

|  | ***pt No*** | ***OS*** | ***p-value*** | ***LC*** | ***p-value*** | ***PFS*** | ***p-value*** |
| --- | --- | --- | --- | --- | --- | --- | --- |
| **Age (years old)** |  |  |  |  |  |  |  |
| ***> 65*** | 17 | 16 | 0.26 | NA | 0.69 | 8 | 0.61 |
| ***≤ 65*** | 14 | 32 |  | 31 |  | 18 |  |
| **Sex** |  |  |  |  |  |  |  |
| ***Male*** | 27 | 22 | 0.06 | 46 | 0.81 | 8 | 0.11 |
| ***Female*** | 4 | 54 |  | 39 |  | 33.5 |  |
| **WHO Performance status** |  |  |  |  |  |  |  |
| ***0*** | 13 | 12 | 0.3 | 47 | 0.68 | 9 | 0.87 |
| ***1*** | 14 | 32 |  | 46 |  | 11 |  |
| ***2*** | 4 | 30 |  | 8 |  | 8 |  |
| **Gross residual tumors** |  |  |  |  |  |  |  |
| ***+*** | 12 | 30 | 0.46 | 31 | 0.62 | 8 | 0.30 |
| ***-*** | 19 | 22 |  | NA |  | 20 |  |
| **Pathology** |  |  |  |  |  |  |  |
| ***Epithelioid*** | 18 | 32 | 0.61 | NA | 0.01 | 11 | 0.38 |
| ***Others*** | 13 | 27 |  | 12 |  | 7 |  |
| **Asbestos exposure** |  |  |  |  |  |  |  |
| ***+*** | 17 | 24 | 0.42 | NA | 0.35 | 9 | 0.79 |
| ***-*** | 7 | 27 |  | 31 |  | 8 |  |
| ***Unknown*** | 7 | 54 |  | 12 |  | 12 |  |
| **Stage** |  |  |  |  |  |  |  |
| ***I or II*** | 21 | 22 | 0.60 | 8 | 0.03 | 6.5 | 0.01 |
| ***III*** | 10 | 32 |  | NA |  | 12 |  |
| **T-stage** |  |  |  |  |  |  |  |
| ***1 or 2*** | 14 | 32 | 0.55 | 14 | 0.03 | 14 | 0.01 |
| ***3*** | 13 | 30 |  | 13 |  | 13 |  |
| ***4*** | 4 | 19 |  | 4 |  | 4 |  |
| **N-stage** |  |  |  |  |  |  |  |
| ***0*** | 21 | 24 | 0.58 | NA | 0.04 | 20 | 0.17 |
| ***1 or 2*** | 10 | 30 |  | 11 |  | 11 |  |
|  |  |  |  |  |  |  |  |
| **Chemotherapy** |  |  |  |  |  |  |  |
| ***+*** | 24 | 27 | 0.82 | 46 | 0.29 | 24 | 0.10 |
| ***-*** | 7 | 30 |  | NA |  | 7 |  |
| **Hyperthermia** |  |  |  |  |  |  |  |
| ***+*** | 8 | 32 | 0.76 | 46 | 0.51 | 11 | 0.60 |
| ***-*** | 23 | 27 |  | 31 |  | 8 |  |
| **Experienced** |  |  |  |  |  |  |  |
| ***≥ 3*** | 19 | 32 | 0.77 | 47 | 0.47 | 11 | 0.53 |
| ***< 2*** | 12 | 24 |  | 17 |  | 8.5 |  |
| **Mediastinal irradiation** |  |  |  |  |  |  |  |
| ***+*** | 14 | 36 | 0.25 | 31 | 0.81 | 14 | 0.31 |
| ***-*** | 17 | 27 |  | NA |  | 17 |  |
| **Surgical tract irradiation** |  |  |  |  |  |  |  |
| ***+*** | 20 | 32 | 0.46 | 46 | 0.41 | 20 | 0.64 |
| ***-*** | 11 | 24 |  | NA |  | 11 |  |

pt: patient, OS: median overall survival from the first treatment (months), LC: median local control from radiotherapy (months), PFS: median progression-free survival from radiotherapy (months), NA: not applicable
